# Supplementary material for: Learning curve and surgical outcome of robotic assisted colorectal surgery with ERAS program
Source: Sci Rep. 2022 Nov 29;12:20566. doi: 10.1038/s41598-022-24665-w (PMC9709162; doi:10.1038/s41598-022-24665-w)
Supplement: Supplementary file 1 — Supplementary Information 1. [file 41598_2022_24665_MOESM1_ESM.zip › LSC Raw data-Ñ[▒K/145/N_ñw╝╨░O▒Kñσ.pdf]

W52 / W52

臺中榮民總醫院
手術室護理記錄

[redacted]

基本資料

- 室別： 二醫手術室-31室，手術科別： CRS，手術類別： 常規，日期： 2021/11/29
- 進入時間： 1300 ； 麻醉開始時間： 1320 ； Time Out時間： 1405 ； 劃刀時間： 1406 ； 手術結束時間： 1900 送出時間： 1910 ；
- 麻醉方式： GE
  診斷：Malignant tumor of rectum
  手術名稱：Laparoscopic Anterior resection and anastomosis - malignant
  術前預防性抗生素：
  - 抗生素名稱 ： Veterin 1GM IVD /AR
  - 時間： 1350
  - 上次簽章: 許晏妮
  ，追加預防性抗生素：
- 手術醫師： 蔣鋒帆，林陳彥，林志安，蔡濬樟
- 麻醉醫師： VS 張思玲
-
  - 刷手護士A ： 彭馨慧，時間： 1300，接替刷手一： 朱詠暄，時間： 1500，接替刷手二： 吳明柔，時間： 1730
  - 流動護士A ： 許晏妮，時間： 1300，接替流動一： 王毓均，時間： 1500，接替流動二： 鄭宜芳，時間： 1730

皮膚狀況

A-V Shunt： 無
術前： 完整
記錄者： 上次簽章: 許晏妮
術後： 完整
記錄者： 上次簽章: 鄭宜芳

手術準備

手術臥式 單一手術臥式

-
  - 手術臥式： 仰臥
  - 使用附件： 溫毯 38 ℃，矽膠枕頭，腳架
  - 皮膚準備： 10%Aq Betadine，Hibiscrub，2% Chlorhexidine
  - 電燒： 使用電燒: 電極片位置： 右大腿，記錄者： 上次簽章: 許晏妮

止血帶T： 無

植入物

- 植入物名稱/號碼/數量/部位：LIGA CLIP 12MM/CI4760003076/20釘/IN PELVIC
  ARTICULATING ENDOSCOPIC LINEAR CUTTER-60 (340mm)/貳釘/IN PELVIC
  STAPLER CIRCULAR 29MM(CDH29A)/壹槍/腸吻合處
  SurgiWrap /100mm*130mm/壹/腹腔

, 記錄者： 上次簽章: 許晏妮

標本送檢

- 細菌培養 無
- 細胞學 無
- 冰凍切片 無
- 病理標本
  組織名稱一：peritoneum
  ,
  組織名稱二：rectum
  ,
  distal cut end

組織名稱三：
，更多組織名稱
    - 組織名稱四：IMA Root
- 截肢 無

標本交付 標本： 4 袋 ， 標本： 0 盒 ， 標本： 0 瓶 ， 細胞學： 0 管 ， Culture Tube： 0 管 ， 被交付醫師： R 林志安，
- 記錄者： 上次簽章: 鄭宜芳
- 時間： 1810

傷口術後敷料

傷口敷料：　　傷口敷料： 無

- 請於圖上標示部位：

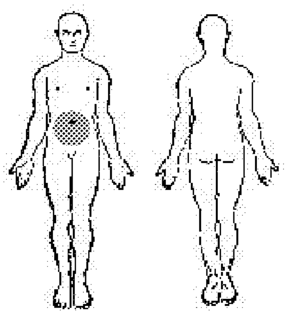

腹部伍切口

- 傷口敷料： MEPORE

導管

導管	天數	導管資訊	插入日期	插入時間	更換日期	拔除日期	拔除時間	拔除原因	預計拔除/更換	預計拔除/更換日期
Penrose Drain(1)	1	號碼:12 mm.; 部位:骨盆腔;	2021/11/29	1810						
A-line (1)	1	號碼:20 號; 部位:左手橈動脈;	2021/11/29	1340						
NG Tube(1)	1	號碼:16 Fr.; 部位:胃;	2021/11/29	1330						
Foley(1)	1	號碼:16 Fr.; 部位:膀胱;	2021/11/29	1345						

用物計數

- 手術中出血量： minimal c.c.
- 手術中尿量： 350 c.c.
-
    - 其它： 無

用物計數： 多切口同時縫合
- 1.器械： 正確，2.縫針： 正確，3.刀片： 正確，4.紗布： 正確，5.腹部墊： 未使用，6.紗球： 未使用，7.PEANUTS ： 未使用，8.紗條： 未使用，9.絲帶： 未使用，10.針頭： 未使用，11.Vessel loop： 未使用，12.Boots： 未使用，13.Jelco： 未使用，14.棉條： 未使用，
    - 第一次：
        - 刷手護士 ： 吳明柔
        - 流動護士： 上次簽章: 鄭宜芳
        - 時間： 1750

，
    - 第二次：
        - 刷手護士 ： 吳明柔
        - 流動護士： 上次簽章: 鄭宜芳
        - 時間： 1800

，
    - 第三次：
        - 刷手護士 ： 吳明柔

- 流動護士： 上次簽章: 鄭宜芳
- 時間： 1820

備註

備註： NIL

- 記錄者： 鄭宜芳 上次簽章: 許晏妮
- 時間： 1920

填寫護理人員 鄭宜芳

正式摘要

W52 / W52

doctorRank:B
